# On-chip deterministic operation of quantum dots in dual-mode waveguides for a plug-and-play single-photon source

**DOI:** 10.1038/s41467-020-17603-9

**Published:** 2020-07-29

**Authors:** Ravitej Uppu, Hans T. Eriksen, Henri Thyrrestrup, Aslı D. Uğurlu, Ying Wang, Sven Scholz, Andreas D. Wieck, Arne Ludwig, Matthias C. Löbl, Richard J. Warburton, Peter Lodahl, Leonardo Midolo

**Affiliations:** 1 Center for Hybrid Quantum Networks (Hy-Q), Niels Bohr Institute, University of Copenhagen, Blegdamsvej 17, 2100 Copenhagen, Denmark; 2 Lehrstuhl für Angewandte Festkörperphysik, Ruhr-Universität Bochum, Universitätsstrasse 150, 44780 Bochum, Germany; 3 Department of Physics, University of Basel, Klingelbergstrasse 82, 4056 Basel, Switzerland

**Keywords:** Optics and photonics, Single photons and quantum effects

## Abstract

A deterministic source of coherent single photons is an enabling device for quantum information processing. Quantum dots in nanophotonic structures have been employed as excellent sources of single photons with the promise of scaling up towards multiple photons and emitters. It remains a challenge to implement deterministic resonant optical excitation of the quantum dot required for generating coherent single photons, since residual light from the excitation laser should be suppressed without compromising source efficiency and scalability. Here, we present a planar nanophotonic circuit that enables deterministic pulsed resonant excitation of quantum dots using two orthogonal waveguide modes for separating the laser and the emitted photons. We report a coherent and stable single-photon source that simultaneously achieves high-purity (*g*^(2)^(0) = 0.020 ± 0.005), high-indistinguishability (*V* = 96 ± 2%), and >80% coupling efficiency into the waveguide. Such ‘plug-and-play’ single-photon source can be integrated with on-chip optical networks implementing photonic quantum processors.

## Abstract (web summary)

Resonantly-excited quantum-dot-based single photon sources feature very high purity, but also limited efficiency due to the need to suppress the residual pump. Here, the authors demonstrate a workaround, performing optical pumping and signal collection in two orthogonal modes inside a nanophotonic circuit.

## Introduction

Photonic quantum information processing encompasses a wide array of emerging quantum technologies including quantum simulators^1–3^, quantum key distribution^4,5^, quantum repeaters^6,7^, and ultimately a full-fledged quantum internet^8,9^. Deterministic generation of coherent single photons is a key building block in realizing these technologies. Quantum dots (QDs) in nanophotonic structures are excellent sources of single photons^10–14^, and planar waveguides are well suited for scaling up to multiple photons and emitters thanks to near-unity photon-emitter coupling^15^ and advanced on-chip functionalities^16^. An ideal single-photon source requires suppressing noise and decoherence, which notably has been demonstrated in electrically contacted heterostructures^17–20^ through resonant optical excitation. However, resonant optical excitation is challenging to implement experimentally as it requires suppressing the excitation laser (same frequency as the QD emission) without affecting the source efficiency.

The conventional approach to pulsed resonant excitation of a QD employs a cross-polarized excitation-collection scheme^10–12^, which inherently limits the collection efficiency of the generated single photons to ≤50%. Recently, elliptical microcavities were proposed and tested to overcome this limit on efficiency^14^, although this method is complicated by the need of controlling two narrow-band cavity resonances relative to the QD. In comparison, planar nanophotonic waveguides offer broadband and robust operation and are naturally suited for efficient laser suppression since the excitation laser and the collection mode can be spatially separated, allowing to construct devices with near-unity generation efficiency. However, resonant excitation of planar devices has so far relied on coupling the pump laser through leaky radiation mode^19,21–23^, which results in high alignment sensitivity and possibly uncontrolled specular scattering.

In this work, we demonstrate a tailored nanophotonic circuit that enables resonant pulsed excitation launched through a grating coupler into a waveguide and subsequent out-coupling of highly coherent single photons from the chip with an additional grating coupler. The circuit distributes the excitation laser to the QDs and strongly suppresses the laser in collection, while maintaining >80% coupling efficiency of single photons. Importantly, as the laser is distributed through the waveguide mode, any QD coupled to the waveguide can be excited by the laser. The circuit realizes an input–output building block that could either be operated as a single-photon source by fiber coupling or integrated as a circuit element in a multiport optical network. The latter is a drop-in replacement of the commonly employed probabilistic photon sources in advanced photonic quantum processors^3,24^, which will greatly benefit the scaling up towards multiphoton and multiemitter quantum information processing^25,26^.

## Results

### Operational principle of the single-photon source circuit

**Fig. 1:**
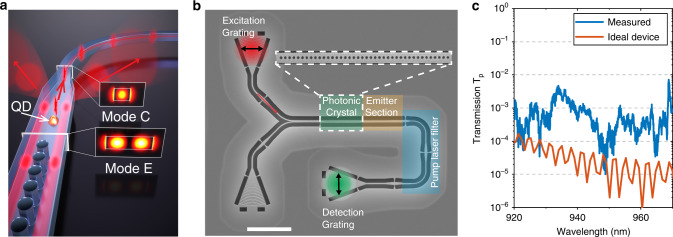
Waveguide-based excitation scheme. **a** Illustration of the mode filtering operation. The resonant pump laser in the first-order waveguide mode excites the emitter and is subsequently squeezed out of the waveguide in the taper section. The QD emission into the fundamental mode of the waveguide is collected efficiently and guided. The photonic crystal acts a mirror for the fundamental mode, thereby enabling the directional out-coupling of the QD signal. **b** Scanning electron microscope image of the fabricated device (length of scale bar is 10 μm). The excitation and collection spots are highlighted with red and green spots. The Y-splitter is used to excite the fundamental and first-order modes of the waveguide. The photonic crystal (zoomed in the inset to highlight the lattice of air holes) selectively transmits only the first-order mode into the emitter section. The pump laser filter section is composed of a waveguide taper and two 90^∘^ bends to suppress the pump laser. The bottom-left grating is used to align the in-coupling of the laser beam by monitoring the reflected signal from the photonic crystal. **c** The measured and calculated transmission *T*_p_ spectrum of the device for a laser coupled in at the excitation grating and collected at the detection grating.

The operational principle of the device is presented in Fig. 1a. We design a two-mode nanophotonic waveguide where the embedded QD is efficiently coupled to the fundamental mode and weakly coupled to the first-order mode. The coupling of the QD to a waveguide mode is quantified through the *β-*factor. By selectively launching the laser into the first-order mode (excitation mode E), the QD is excited and the single-photon emission collected through the fundamental mode (collection mode C). In order to efficiently collect only the single photons, the residual excitation in laser mode E must be filtered out, while ensuring lossless propagation of mode C. An adiabatically tapered waveguide section is employed to satisfy these demands simultaneously. In the taper section, the E mode becomes leaky and is extinguished by the deliberate introduction of sharp waveguide bends. The adiabatic taper ensures the efficient transfer of the mode C into the single-mode regime that subsequently can be coupled into an optical fiber. We furthermore employ a one-dimensional photonic crystal as a backward reflector for single photons propagating in the mode C to maximize unidirectional out-coupling efficiency. A scanning electron microscope image of the nanofabricated device highlighting the three key elements of the device (photonic crystal, two-mode waveguide with emitters, and waveguide taper-based pump laser filter) is shown in Fig. 1b (see Supplementary Note 1 for the fabrication method). Three high-efficiency (>65%) grating couplers^27^ are fabricated for in- and out-coupling of light from free-space to the device.

The input excitation grating is connected to a 300-nm-wide single-mode waveguide, followed by a Y-splitter that launches the excitation laser into both the E and C modes of the two-mode waveguide^28^. The Y-splitter together with the photonic crystal selectively prepares the mode of the excitation pulse (Supplementary Notes 2 and 3). The photonic-crystal section is a key design element of the device serving a dual purpose: (1) as a backward reflector for unidirectional collection of single-photon emission and (2) to selectively prepare the excitation laser in the mode E. It is designed such that it reflects the C mode and transmits the E mode into the emitter section of the waveguide. Figure 1c shows the measured transmission spectrum *T*_p_(*λ*) of the excitation laser through the device, which quantifies how well the residual excitation light can be suppressed. *T*_p_ is extracted by comparing the transmitted laser intensity in two nominally similar devices with and without the photonic-crystal section. For reference, the calculated performance for an ideal device without any fabrication imperfections is shown in Fig. 1c, and remarkably ideal performance with *T*_p_ ~ 10^−5^ is observed in certain wavelength bands. The minor deviations in the measurements from ideal performance can be attributed to an unintentional disorder in the nanofabricated photonic crystal.

### Pre-characterization of the device

In order to assess the performance of the device as a single-photon source, the laser suppression *T*_p_ should be related to the single-photon emission probability. An essential figure-of-merit is the intensity of the residual pump intensity relative to the intensity of the emitted single-photon signal, i.e., the single-photon impurity *ξ*, which is the ratio of the number of laser photons to the single photons. *ξ* is related to the measured second-order coherence function through *g*^(2)^(*τ* = 0) = 2*ξ* − *ξ*^2^^29^. We relate *T*_p_ and *ξ* as follows: The residual laser intensity at the out-coupling grating is given by *I*_p_*T*_p_, where *I*_p_ is the input pump laser intensity. Under pulsed resonant excitation, we express the single-photon intensity at the collection grating as *I*_sp_ ≈ *β*_E_*β*_C_*I*_p_/2, which is a simplified expression for clarity that holds below saturation of the QD and when omitting any effect of dephasing. The factor of 1/2 accounts for the power splitting of the excitation laser into the modes E and C at the Y-splitter. Supplementary Note 8 details the complete theory without these restrictions. *β*_E_ and *β*_C_ are the photon *β*-factors^15^ expressing the probability of the QD to absorb a pump photon and emit a single photon into the waveguide, respectively. Consequently we have

1 $$\xi =\frac{{I}_{{\rm{p}}}{T}_{{\rm{p}}}}{{I}_{{\rm{sp}}}}=\frac{2{T}_{{\rm{p}}}}{{\beta }_{{\rm{E}}}{\beta }_{{\rm{C}}}}.$$

**Fig. 2:**
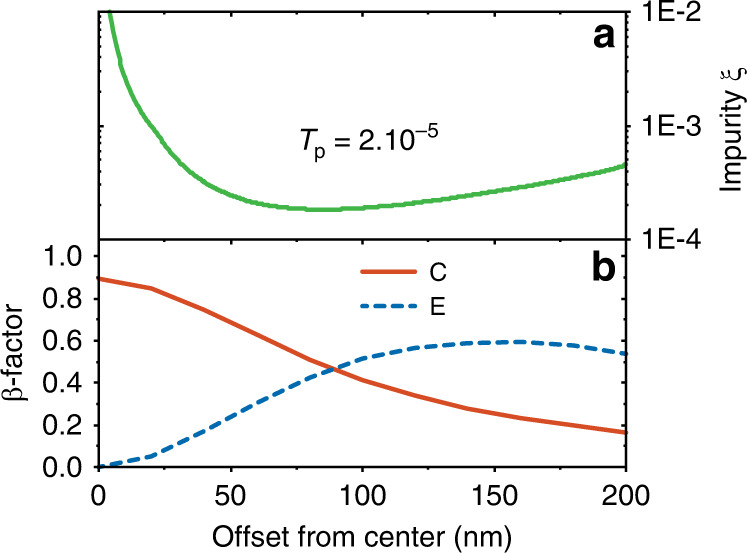
Predicted device performance. **a** Expected single-photon impurity *ξ* for the experimentally achieved value of *T*_p_ = 2 ⋅ 10^−5^ and as a function of different emitter locations in the waveguide. **b** Calculated *β*-factor for the two waveguide modes as a function of the offset distance of the emitter from the center of the waveguide.

The QD position affects the emitter-photon coupling *β*_C_ and *β*_E_ and therefore the value of *ξ*. Figure 2 (bottom panel) shows the calculated *β*-factors as a function of transverse offset from the waveguide center. A QD positioned exactly at the center of the waveguide maximally couples to *β*_C_, but is not pumped by the excitation laser in the mode E as *β*_E_ ~ 0. The optimum QD position that simultaneously minimizes the photon impurity *ξ* and maintains a high *β*_C_ is seen in Fig. 2 for the measured device parameters, (*T*_p_ = 2 ⋅ 10^−5^). For a QD position where a high single-photon coupling efficiency of *β*_C_ ≃ 0.9 can be reached, we obtain *ξ* ≃ 5 ⋅ 10^−4^, which implies that *g*^(2)^(0) ≃ 10^−3^ can be achieved. We note that further reduction in *T*_p_, e.g., by optimizing the filter design or reducing fabrication disorder, could lead to even better single-photon purity even when *β*_C_ approaches unity.

From a practical standpoint, the probability of locating well-positioned QDs is dependent on the density of the QDs in the wafer and the length of the emitter section. The wafer employed in our measurements has a QD density of  ≈ 10/μm^2^ with emitter section dimensions of 40 μm  × 0.45 μm. From Fig. 2, we observe that *β*_C_ > 0.5 requires that the QD is located within an offset of 75 nm from the center. Given the spectral inhomogeneity of QDs, we estimate to find around 10 QDs that are well-positioned in the emitter section within the spectral window where *T*_p_ < 10^−4^ (c.f. Supplementary Note 4 regarding QD selection in two devices). We carried out resonance-fluorescence measurements on seven devices and found at least one QD per device with *β*_C_ ≈ 0.8.

### Pulsed resonant excitation of a quantum dot

**Fig. 3:**
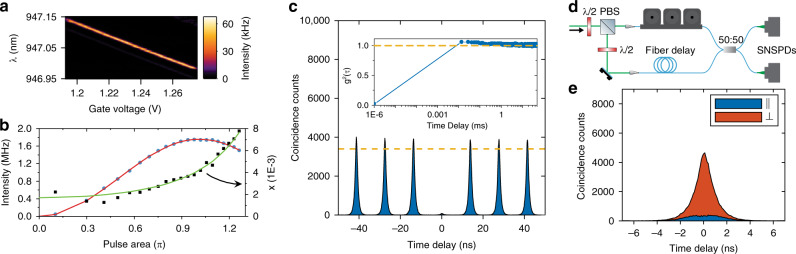
Demonstration of pure and indistinguishable single photons by pulsed deterministic resonant excitation. **a** QD resonance-fluorescence intensity under cw laser excitation at a power of *P* = 0.01 ⋅ *P*_s*at*_, where *P*_s*at*_ is the saturation power. **b** Power dependence of the resonance-fluorescence intensity and the photon impurity *ξ*. The Rabi oscillations (red curve) of the two-level system are modeled including a pure dephasing rate of *γ*_d_ = 0.2 ns^−1^. **c** The intensity-correlation histogram in a Hanbury Brown and Twiss experiment for *π* − pulse excitation. The second-order correlation function *g*^(2)^(0) = 0.02 ± 0.005 is extracted from the fitted amplitude of the central peak relative to the fitted amplitude for peaks at a time delay of 50 μs (dashed line). The inset shows *g*^(2)^(*τ*) measured by integrating the coincidences under the peak over the 50 μs time span. **d** Schematic of the Hong–Ou–Mandel interferometer used for measuring the indistinguishability of two subsequent photons delayed by the laser pulse separation of 13.7 ns. **e** Coincidence counts after the Hong–Ou–Mandel interferometer when the input photons are co-polarized (blue) and cross-polarized (red).

Experimental demonstration of waveguide-assisted pulsed resonant excitation of an optimally coupled QD was demonstrated on the device shown in Fig. 1b. Resonance-fluorescence measurement from a neutral exciton under continuous wave excitation is shown in Fig. 3a, which is carried out to identify QD resonances and demonstrate low-noise performance. We observe distinct QD resonances, free of excitation laser background (*T*_p_ = 2 ⋅ 10^−5^), with a linewidth of 800 MHz that tune with the applied bias voltage. The broadening of the QD resonances beyond the natural linewidth (250 MHz, as estimated from lifetime measurement) occurs primarily due to slow spectral diffusion (time scale of 10 ms), which is not relevant for pulsed operation and could be rectified by active feedback^30,31^. Deterministic pulsed resonant excitation is performed with 26 ps optical pulses tuned to the QD resonance. The observed Rabi oscillations of the detected intensity are shown in Fig. 3b that are modeled as a driven two-level system including minor pure dephasing, see Supplementary Note 8 for details of the model. The single-photon impurity *ξ* was extracted at each excitation power by comparing the detected intensity with the QD tuned on- and off-resonance by using the electrical control. The power-dependent *ξ* reflects the fact that the QD transition saturates when approaching *π*-pulse excitation while the residual laser background scales linearly with pump power, and this behavior is fully captured by the theoretical model, cf. Fig. 3b. The coupling efficiency of the QD emission to the waveguide, quantified through *β*_C_, is extracted by comparing the measured *ξ*(*P* → 0) = 1.7 ⋅ 10^−3^ and *T*_p_ = 2 ⋅ 10^−5^ values with the calculations in Fig. 2. This comparison results in *β*_C_ = 0.8, which corresponds to a QD position offset from center of the waveguide by  ≈20 nm. Hence, the device enables 80% collection efficiency of the single photons into the waveguide while ensuring low laser background. At *π*-pulse, i.e., deterministic QD preparation, we find *ξ* = 0.004 (*g*^(2)^(0) = 0.008). We detect a single-photon rate of 1.8 MHz, which is primarily limited by the collection optics in the device characterization setup and can readily be improved further. Supplementary Note 5 presents a detailed description of the observed source efficiency that fully accounts for the independently measured parameters.

### Single-photon source purity and indistinguishability

Having demonstrated pulsed resonant excitation through the waveguide mode, we proceed to the characterization of the quality of the single-photon source. Figure 3c shows the intensity-correlation histogram measured at *π*-pulse excitation using a Hanbury Brown and Twiss interferometer. A clearly suppressed peak at time delay *τ* = 0 ns is observed that is normalized to the long *τ* limit to extract *g*^(2)^(*τ* = 0) = 0.020 ± 0.005. The observed value of *g*^(2)^(0) is higher than the expected value for the measured device parameters, which can be attributed to the temporal extent of the excitation laser pulses (26 ps) in comparison to the QD decay time (640 ps) that results in non-zero two-photon emission probability^32^. We estimate that excitation laser with  <3 ps pulse width would be required to reach the *g*^(2)^(0) value limited by the device^32,33^. Even better performance could be achieved by reducing *T*_p_ either by design or through an improvement in the fabrication. The current design enables *T*_p_ ≈ 10^−6^ (Fig. 1c) corresponding to *g*^(2)^(0) ≈ 10^−4^, which approaches the best reported value in the literature obtained with two-photon resonant excitation^34^, where pump filtering is not a challenge.

Most applications of single photons in quantum information require high indistinguishability of the photons, which we measure in a Hong–Ou–Mandel (HOM) experiment by interfering two subsequently emitted photons in an unbalanced fiber-based Mach–Zehnder interferometer, cf. Fig. 3d. Figure 3e shows the recorded correlation histogram between the two detectors, where the strong suppression of coincidences for zero detector time delay testifies the high degree of indistinguishability of the emitted photons. By controlling the polarization of the incoming photons, the reference case of fully distinguishable photons (perpendicular polarization case) is recorded and we extract the HOM interference visibility *V* that quantifies the photon indistinguishability. We measure a raw visibility of *V*_raw_ = (91 ± 2)%, which, after correcting for the finite *g*^(2)^(0) and setup imperfections corresponds to *V* = (96 ± 2)% (see Supplementary Note 6 for details). The measured indistinguishability is on par with the best reported value with cross-polarized resonant excitation^10^ and only superseded by experiments relying on excitation pulse-engineering^35,36^.

## Discussion

In conclusion, we have experimentally demonstrated an efficient waveguide circuit for deterministic pulsed resonant excitation of QDs embedded in planar photonic nanostructures. The circuit enables the realization of an efficient “plug-and-play” single-photon source featuring near-unity single-photon coupling, as well as high purity and indistinguishability. The robust excitation process implies that the device could be operated continuously without any realignment, and as a proof-of-concept we operated the source hands-free for over 110 h with <2% fluctuation in the generation rate (Supplementary Note 7). The circuit will also enable improving the collection efficiency for more advanced excitation schemes relying on dichromatic laser pulses^36^, which are typically limited by low-efficiency spectral filters. Large Purcell enhancement of the radiative decay rate for overcoming residual decoherence and increasing the source repetition rate can also be achieved through a small modification of the circuit^37^. The modified circuit includes an additional photonic crystal (same parameters as the first one) after the emitter section to form a standing-wave cavity for QD emission in mode C. An obvious next step is to implement direct chip-to-fiber coupling^38^ thereby circumventing the loss associated with collection, mode shaping and subsequent fiber coupling. Another opportunity is to scale-up the circuit so that one excitation pulse could be pumping multiple QDs in parallel. In order to overcome the spectral inhomogeneity of QDs, such a device will require independent tunability of the different QDs to match the frequency of the excitation pulse^39,40^. With such an approach with the circuit, the benefits of the scalable planar platform will be fully exploited in the ongoing pursuit of scaling up single-photon technology^41^.

## Methods

### Experimental setup

In order to perform single-photon generation experiments, the sample is cooled to a temperature of 1.6 K in a closed-cycle cryostat with optical and electrical access. The excitation laser and the QD emission are focused and collected at the respective grating outcouplers (Fig. 1b) using a wide field-of-view microscope objective. A 20:80 (reflection:transmission) beam splitter is used to separate the excitation and collection into separate optical paths, with the high-efficiency path used for collection. The collected single-photon emission is coupled into a single-mode optical fiber and sent through a spectral filter constituting of an etalon (linewidth = 3 GHz; free spectral range = 100 GHz). The spectrally filtered single-photon stream can be directed to either a compact fiber-based unbalanced Mach–Zehnder for measuring two-photon interference or directly to a super-conducting nanowire single-photon detector (SNSPD). The gate voltage across the QD is tuned using a low-noise voltage source with an RMS noise  <50 μV, which corresponds to  <0.1Γ, where Γ is the linewidth of the QD.

### Samples

A scanning electron microscope image of the nanofabricated device with a footprint of 50 × 45 μm^2^ is shown in Fig. 1b. The photonic-crystal section is a one-dimensional lattice of 40 air holes with radius of 70 nm and lattice spacing of 210 nm. The emitter section (450-nm-wide and 170-nm-thin suspended GaAs nanobeam waveguide) supports the two propagating modes E and C. Self-assembled indium arsenide (InAs) QDs, embedded in a p-i-n diode (see Supplementary Fig. 1 for details), are randomly located across the waveguide with an average density of 10/μm^2^. This density is high enough to comfortably find several QDs within the best laser suppression windows in all 20 fabricated devices. The suspended waveguide is electrically contacted (contacts not shown in the figure) to tune the QDs and to suppress noise leading to spectral drift. The pump laser filter is a 5-μm-long linear taper, which gradually reduces the waveguide width from 450 to 200 nm. Two consecutive 90° waveguide bends are introduced to further extinguish the weakly-guided E mode. Three shallow-etched grating couplers are fabricated for in- and out-coupling of light from free-space to the waveguides. These gratings enable  >65% collection efficiency of light in the C mode from the waveguide into a single-mode optical fiber^27^.

## Supplementary information

Supplementary Information

## Data Availability

The data are available from the corresponding authors upon reasonable request.

## Code availability

COMSOL Multiphysics was used for designing the nanostructure. Data analysis was performed using MATLAB. The scripts are available from the corresponding authors upon reasonable request.
