# Supplementary material for: On-chip deterministic operation of quantum dots in dual-mode waveguides for a plug-and-play single-photon source
Source: Nat Commun. 2020 Jul 29;11:3782. doi: 10.1038/s41467-020-17603-9 (PMC7391626; doi:10.1038/s41467-020-17603-9)
Supplement: Supplementary file 1 — Supplementary Information [file 41467_2020_17603_MOESM1_ESM.pdf]

Supplementary Information - On-chip deterministic operation of quantum dots in dual-mode waveguides for a plug-and-play single-photon source

Uppu et al.

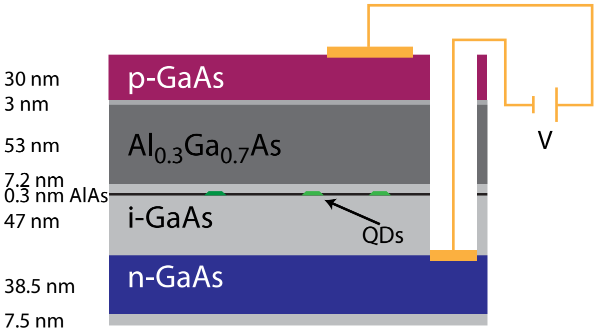

Supplementary Figure 1. Outline of p-i-n diode heterostructure used to realize the device.

SUPPLEMENTARY NOTE 1. HETEROSTRUCTURE COMPOSITION AND SAMPLE FABRICATION

The samples are fabricated on a GaAs membrane grown by molecular beam epitaxy on a (100) GaAs substrate. A 1150-nm-thick $Al_{0.75}Ga_{0.25}As$ sacrificial layer is used to isolate and suspend the membrane from the substrate after wet etching. The membrane structure is shown schematically in Supplementary Fig. 1. It contains a layer of self-assembled InAs quantum dots (QDs) grown with a technique that removes the electron wetting layer states [1], embedded in a p-i-n diode for the reduction of charge noise and control of the charge state and Stark tuning of the emitter. A 53-nm-thick $Al_{0.3}Ga_{0.7}As$ layer is used as a barrier to limit the current to a few nA when the diode is operated under forward bias.

Electrical contacts to the p-doped and n-doped layers are fabricated first. Reactive-ion etching (RIE) is used to open vias to the buried n-layer and Ni/Ge/Au contacts are deposited by electron-beam physical vapor deposition. The contacts are annealed at 430 °C. Subsequently Cr/Au pads are deposited on the surface to form Ohmic p-type contacts. The waveguides are patterned using electron-beam lithography at 125 keV (Elionix F-125) and etched in the GaAs membrane by inductively-coupled plasma RIE in a $BCl_3/Cl_2/Ar$ chemistry. The sample is then undercut and cleaned following the procedure explained in Ref. [2].

SUPPLEMENTARY NOTE 2. DESIGN OF THE PHOTONIC CRYSTAL SECTION

We employ a multimode photonic crystal nanobeam waveguide in the device to prepare the excitation laser in mode E and as backward reflector for single photons in mode C. The band structure for the multimode photonic crystal nanobeam waveguide (width = 450 nm) is shown in Supplementary Fig. 2(a) where k_z is the projected wave-vector for propagation along the nanobeam waveguide. The one-dimensional photonic crystal is realized as an array

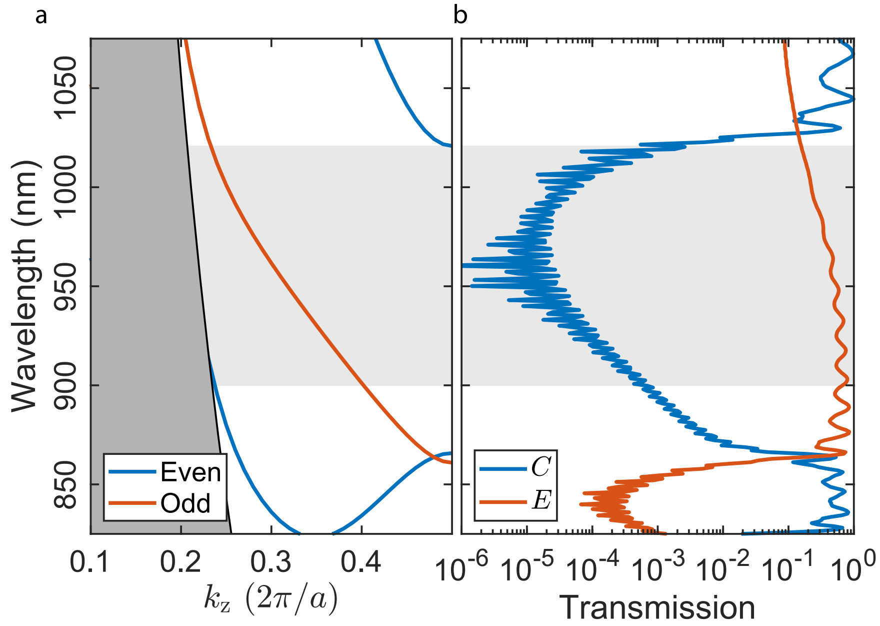

Supplementary Figure 2. (a) Photonic bandstructure calculated for the multimode photonic crystal waveguide shows the behavior of the first odd and even modes allowed in the waveguide. The odd mode is labeled as E as its employed to excite the quantum dot and the even mode is label C as its employed to collect the QD emission. The dark gray shaded region is above the light line of the 170 nm thin slab. (b) Calculated transmission of the E and the C modes across a 20-hole photonic crystal waveguide section of 450 nm width, 170 nm thickness, and hole-to-hole distance (lattice constant) of 210 nm. The light gray shaded region in both the figures corresponds to the stop gap of an infinite photonic crystal.

of circular air holes with a radius of 70 nm and a hole-to-hole spacing of 210 nm. The solid curves below the light line (dark gray area) indicate the propagating modes confined in the nanobeam waveguide and are color coded according to their transverse spatial symmetry. In the wavelength region highlighted in light gray, the photonic crystal supports a stop gap for even modes while allowing partial transmission of odd symmetry modes. In our design, we couple the resonant pump laser into the odd mode that excites the QDs (labeled as excitation mode E) and collect the single photon emission through the even mode (labeled as collection mode C). Under ideal conditions (lossless and infinitely long photonic crystal), light coupled to mode C is completely reflected, while that in mode E would be transmitted.

We employ finite-difference time-domain (FDTD) calculations of a 20-hole photonic crystal nanobeam waveguide to investigate the performance of a finite-length device. The results from the calculations are shown in Supplementary Fig. 2(b). The excitation mode E is attenuated by roughly 50% over the spectral band of interest for the QDs (i.e. between 920–960 nm). The mode C is instead extinguished by more than a factor of 10^4 near the center

of the band gap. Imperfections introduced during fabrication can reduce the total mode suppression. For this reason, the fabricated device has been designed with 40 holes.

In the transmission of the laser, this high degree of suppression of the C mode by the photonic crystal filter allows preparing the resonant excitation laser selectively in the E mode. In the collection of QD emission, the photonic crystal acts as a perfect (>99.99% reflectivity) mirror for single photons coupled to the C mode, thereby enabling unidirectional collection.

SUPPLEMENTARY NOTE 3. GENERATION OF THE EXCITATION MODE

To generate the odd mode in the dual-mode waveguide, we use a (50/50) power divider implemented with a Y-shaped waveguide junction. When light is funneled from one of the two branches of such Y-splitter, it produces, by reciprocity, a nearly equal superposition of the even and odd mode in the dual-mode waveguide. From finite-element calculations, we find that 50% of the light is coupled into the odd mode, 46% is coupled into the even mode, and the rest is scattered off-chip or reflected into the other branch. The fraction of laser power coupled into the even mode is subsequently reflected (> 99.99%) by the photonic crystal as discussed in Supplementary Note 2, whereas the odd mode goes through with a small loss (< 40%; c.f. Supplementary Fig. 2(b)) and is used for the excitation of the QDs in the emitter section. Under pulsed resonant excitation of the QD, an energy per pulse of approximately 10 fJ (corresponding to a π-pulse area) enters the Y-splitter. From numerical simulations of the Y-splitter and photonic crystal we estimate that 3 fJ (14000 photons per laser pulse at 947 nm) of excitation laser energy reaches the excitation mode at the QD position.

SUPPLEMENTARY NOTE 4. RESONANCE FLUORESCENCE OF QUANTUM DOTS

The optical setup for exciting and collecting the emission from QDs is shown in Supplementary Fig. 4, which is described in the next section. One important distinction during the characterization phase of the circuit is the absence of spectral filtering, i.e. the etalon in Supplementary Fig. 4 was removed during this step to enable searching of QDs over a very wide frequency span. A tunable continuous-wave narrow-bandwidth diode laser is coupled into the circuit through the input grating. The signal at the output grating is coupled out into a fiber-coupled superconducting nanowire single-photon detector (SNSPD), without any spectral filtering, i.e. etalon in Supplementary Fig. 4 is absent in this step. The continuous-wave laser power coupled into the grating was ≈ 50 nW. At a gate voltage $V_g = 1$ V, where no charge states can be populated in the QD, the output signal is recorded by continuously tuning the laser wavelength to estimate the transmittance of the excitation

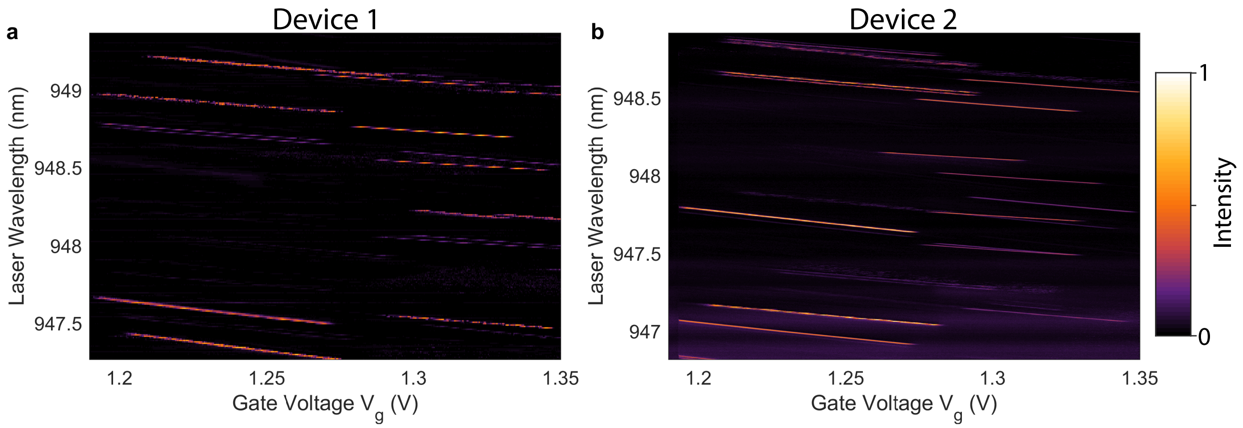

Supplementary Figure 3. Wide range (≈ 2 nm) spectral search for quantum dots in two devices. Resonance fluorescence from two nominally similar devices are shown in a) and b) in the spectral band with $T_{\mathrm{p}} < 10^{-4}$. The excitation laser in incident at the input grating and the signal at the output grating is measured using a superconducting nanowire single-photon detector (SNSPD). The output signal is registered at every combination of excitation laser frequency and the gate voltage V_{g} across the QDs. Distinct fluorescence lines are evident in both the devices across the entire spectral window indicating that the device suppresses the excitation laser in comparison to the QD fluorescence.

laser T_{p} across the circuit. Figure 1(c) of the main text shows the measured T_{p} for one such circuit. We measured the laser transmission across 20 different devices and found that each one supported a spectral window of ≈ 5 nm with $T_{\mathrm{p}} < 10^{-4}$, which was sufficient for achieving low single-photon impurity ξ.

Subsequently, we search for QDs coupled to the waveguide by performing a wavelength-voltage sweep and recording the signal at the output. The wavelength span of the sweep was typically centered in the spectral window with $T_{\mathrm{p}} < 10^{-4}$. The gate voltage V_{g} span of the sweep was between 1.19 V and 1.35 V with a very fine step size of 0.2 mV. Supplementary Figure 3 shows the measured output signal on two of the seven devices that we investigated. Each distinct bright line is the resonance fluorescence from a single QD. Distinct Coulomb blockade charge plateaus are observed with strongly suppressed laser background across the entire range. The emission wavelength of a single QD resonance can be electrically tuned through Stark effect by 0.2 nm. We identify that the QD resonances observed between 1.2 V and 1.28 V are the neutral excitons and those above are the negatively charged excitons. We note that the charged and the neutral excitons captured in the 2 nm spectral window belong to different QDs as the typical binding energy difference between the neutral and singly-charged exciton was ≈ 4 nm, measured across several QDs in the wafer. Therefore, we find around 15 QDs that are clearly coupled to the waveguide with low ξ and high β_{C}. If we restrict to the neutral excitons, which possess linear dipoles, it is easy to locate at least

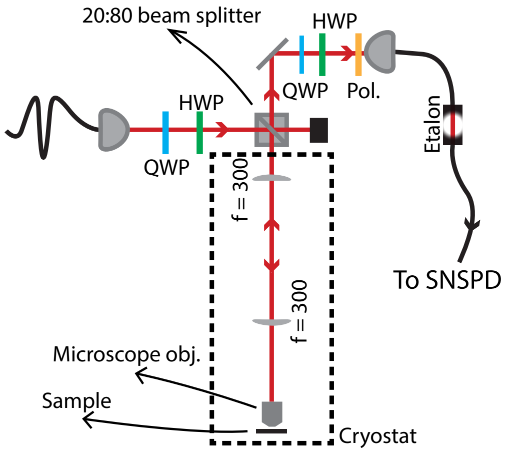

Supplementary Figure 4. Schematic of the optical setup used in the excitation and collection of emission from a QD embedded in the nanophotonic device. The sample is cooled to a temperature of 1.6 K in a closed-cycle cryostat. A set of quarter (QWP) and half (HWP) wave plates are used to control the polarization of the incident and collected light.

5 QDs that could potentially be employed as single-photon sources. The apparent better quality (smoother lines) in Supplementary Fig. 3(b) is due to the smaller wavelength step of 1.5 pm in comparison to the 9 pm step size in (a). The QD employed in the measurements in this article is the neutral exciton observed in the bottom left corner of resonance fluorescence map of Device 2.

SUPPLEMENTARY NOTE 5. SOURCE EFFICIENCY

The optical setup employed in our experiments is shown in Supplementary Fig. 4. The transmittance of each optical element used in the setup is carefully characterized using a continuous-wave narrow bandwidth diode laser operating at 947.1 nm. The complete breakdown of the source efficiency into the collection and QD efficiencies is presented in Supplementary Table 1. A resonant excitation laser is collimated and imaged to the back focal plane of a low-temperature compatible microscope objective (NA = 0.81). The microscope objective couples the laser light into the excitation grating coupler as well as collects the QD emission at the grating outcoupler. The resonant laser and the collected emission is separated into different spatial modes using a 20:80 (reflection:transmission) beam splitter, where the transmission arm is used for collection. The collected emission passes through a set of quarter and half wave plates (QWP, HWP in the figure) and is imaged onto a fibre collimator. The collection efficiency of the imaging system T from the device to the entrance of the collection fibre is $51 \pm 2\%$. The QD emission coupled into the waveguide is fibre-coupled using the grating outcoupler-fibre relay. In the current setup, the mode-matching efficiency of the grating outcoupler to the fibre η_f is limited to $24 \pm 2\%$, which is significantly lower

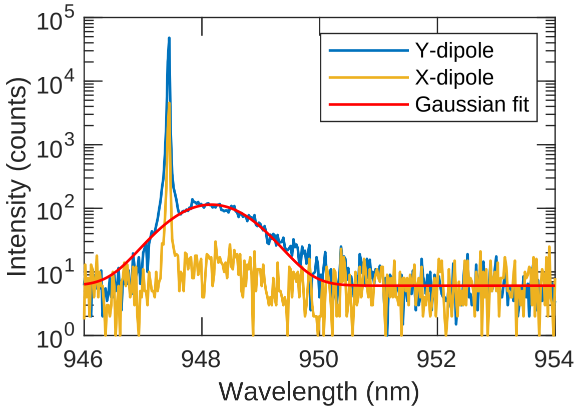

Supplementary Figure 5. Spectrally-resolved resonance fluoresence of the QD excited using a narrow bandwidth diode laser. The emission spectra are collected at an excitation power of 1 nW and gate voltage of 1.235V with the laser tuned to $\lambda = 947.075$ nm (X-dipole; yellow curve) or 947.108 nm (Y-dipole; blue curve). The red curve is the Gaussian fit of the emission in the photon side band when exciting the Y-dipole.

than the $> 65\%$ reported in our earlier work [3]. This is only limited by an unoptimized image relay line in the current setup that could readily be improved by a proper lens choice. In a recent work, we demonstrated that the grating outcoupler efficiency can be improved to $> 82\%$ (see Appendix D.3 of Ref. [4]) by including a distributed Bragg reflector below the GaAs membrane. The chip-to-fiber efficiency can be improved even further to $> 96\%$ by replacing grating outcouplers with tapered-waveguide coupling into tapered optical fibers. [5–7]

In the following, we analyze the emission efficiency of the QD employed in the measurements taking fully into account all relevant loss processes. We operate the QD at a gate voltage of 1.235 V, which ensures selective excitation of the neutral exciton X_0. X_0 has two bright states from spectrally non-degenerate dipoles (fine structure splitting = 10 GHz) with orthogonal linear polarization. The transverse location of QD in the waveguide determines the coupling of the dipoles to the waveguide modes. The coupling asymmetry of the two dipoles is estimated from the ratio of the resonance fluorescence intensities at a fixed weak excitation power ($\approx 1\%$ of the saturation power for the well-coupled Y-dipole). The spectrally-resolved emission with the excitation laser on resonance with X- and Y-dipoles is shown in Supplementary Fig. 5. Under pulsed resonant excitation, both the dipoles are driven by the broad spectral bandwidth (40 GHz) of the pulse, and the coupling asymmetry relates to the emission asymmetry in the two dipoles. By integrating the area under the spectrum, we estimate that the emission fraction into the well-coupled Y-dipole is $\eta_Y = 91 \pm 1\%$. The resonance fluorescence spectrum in Supplementary Fig. 5 also exhibits a weak pedestal, which corresponds to the residual phonon side band. The phonon sideband is

fitted to a Gaussian to estimate the fraction emitted outside the zero phonon line, $1 - \eta_{\mathrm{ZPL}} = 8.5 \pm 1\%$. The loss into the phonon side-band can be reduced using one of two methods: 1) Lowering the sample temperature [8] and 2) Purcell enhancement of radiative decay rate [9]. The latter can be implemented by incorporating a second photonic crystal after the emitter section to realize a cavity. However, such a device will require frequency tuning of the cavity for broadband operation.

Apart from these radiative losses outside the zero phonon line of the Y-dipole, the QD neutral exciton can weakly couple to non-radiative dark state. This contribution is obtained by modeling the weak bunching observed (cf. data in Fig. 3(c) in the main text) of maximum amplitude $\max(g^{(2)}(\tau))/g^{(2)}(\tau \to \infty) = 1.03$ with an exponential decay rate of 0.25 μs^{-1} using a 3-level system and extracting the dark state population [10, 11]. The resulting probability for the QD to blink into the dark state is $1-\eta_{\mathrm{blink}} = 3\%$. The product of η_{Y}, η_{ZPL}, and η_{blink} is the intrinsic efficiency of the QD, which is $80 \pm 1\%$. The coupling efficiency of the QD emission in Y-dipole to the waveguide collection mode C, quantified through β_{C}, is found to be $80 \pm 5\%$, as discussed in the main text. The collected emission is spectrally filtered using an etalon filter with a linewidth of 3 GHz (peak transmission efficiency $\eta_{\mathrm{s}} = 80 \pm 1\%$) centered at the Y-dipole emission wavelength so as to filter out the phonon side band and the X-dipole. The filter efficiency η_{s} can be increased to $> 95\%$ using a tunable air-spaced cavity instead of the solid glass etalon, which overcomes the absorption loss in the glass. The total end-to-end efficiency of the source is $\eta_{\mathrm{Y}}\eta_{\mathrm{ZPL}}\eta_{\mathrm{blink}}\beta_{\mathrm{C}}\eta_{\mathrm{p}}T\eta_{\mathrm{f}}\eta_{\mathrm{s}}$, where all the measured contributions to propagation loss from source to detector are listed in Supplementary Table 1. Specifically the minor residual loss inside the device due to propagation in the waveguides was measured by dedicated transmission measurements through waveguides of varying lengths. The estimated propagation loss in the waveguide is 10.5 dB/mm, which for the $\approx 100\mu$m long device results in a loss (1-η_{p}) of $\approx 15 \pm 5\%$. The overall efficiency of the source was found to be 5.3%, and the complete break-down of the efficiency lays out straightforward path ways to improve this further. The detected and expected photon count rates listed in the table take into account the detector deadtime of 100 ns. Notably the detected rate of single photons match the expected rate to within the error bars of the measured parameters, emphasizing the full quantitative understanding of the device.

SUPPLEMENTARY NOTE 6. ANALYZING INDISTINGUISHABILITY DATA AND SETUP PARAMETERS

We employ the procedure discussed in Ref. 12 and correct the raw indistinguishability for setup imperfections and finite $g^{(2)}(0)$. The raw coincidence counts, shown in Fig. 3(e) of the main text, are fitted with double sided exponential functions convoluted with the measured instrument response function of the detectors. To account for the low background count, we employ a Poissonian noise model with amplitudes of the central peak A_0 and the

QD efficiency	Y-dipole fraction (η_{Y})	$91 \pm 1\%$
	Filtered phonon sideband ($1 - \eta_{\mathrm{ZPL}}$)	$8.5 \pm 0.5\%$
	QD blinking ($1 - \eta_{\mathrm{blink}}$)	3%
	β−factor (β_{C})	$80 \pm 5\%$
Collection efficiency	On-chip propagation loss ($1 - \eta_{\mathrm{p}}$)	$15 \pm 5\%$
	Collection optics (T)	$51 \pm 2\%$
	Grating-to-fibre collection (η_{f})	$24 \pm 2\%$
	Spectral filter (η_{s})	$80 \pm 1\%$
Total efficiency		$5.3 \pm 0.7\%$
Detection efficiency		$65 \pm 5\%$
	Laser rep. rate	72.5 MHz
	Expected rate	2.5 ± 0.4 MHz
	Detected rate	2.2 MHz

Supplementary Table 1. This table presents the end-to-end efficiency of the single photon source.

peak at long time delay A_∞[13]. The fitted peak amplitude at zero-delay A_0 is rescaled to A_∞. This procedure is carried out to extract the normalized central peaks $A_\perp$ and $A_\parallel$ for co- and cross-polarized photons, respectively. The normalization procedure corrects for systematic variations in the total count rates that could occur when switching between the two configurations. The normalized areas are related to the raw indistinguishability V_{raw} as

$$V_{\mathrm{raw}} = \frac{A_\perp - A_\parallel}{A_\perp}. \quad (1)$$

For an intrinsic indistinguishability of V the expected amplitude of the central peak accounting for setup imperfections is given by

$$A(V) = \left(R^3T + RT^3\right)[1 + 2g^{(2)}(0)] - 2R^2T^2(1-\epsilon)^2V, \quad (2)$$

where R and T is reflectivity and transmission of the beam splitter, and $(1-\epsilon)$ is the classical visibility of the interferometer. Using Supplementary Eq. (1) and (2), we can estimate the instrinsic visibility V from V_{raw} using the relation

$$V = \frac{[1 + 2g^{(2)}(0)]\left(R^2 + T^2\right)V_{\mathrm{raw}}}{2RT(1-\epsilon)^2}. \quad (3)$$

In our experiment, we measured $R = 0.476$, $T = 0.524$, and $g^{(2)}(0) = 0.02 \pm 0.005$, $V_{\mathrm{raw}} = 0.91 \pm 0.02$, and $(1-\epsilon) > 0.99$. These values for the setup parameters results in an intrinsic visbility of $V = 0.97 \pm 0.03$.

We also employed an alternative approach to correct the HOM visibility. In the setup, we employ a quarter-wave plate and a linear polarizer in the collection path to optimally collect

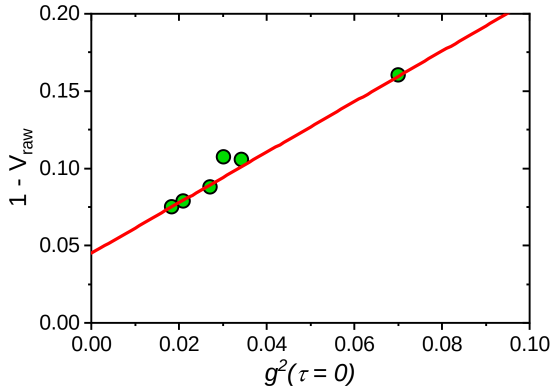

Supplementary Figure 6. Relation betwen the QD signal to laser extinction for extracting the photon indistinguishability. At a constant excitation power, laser extinction was tuned by increasing the background scatter at the collection fibre. The plot shows measurements of $g^{(2)}(0)$ and the HOM visibility V_{raw} at a given laser extinction (circles). The y-intercept of the linear fit (red curve) to the data is used to estimate the intrinsic HOM visibility, which is found to be $V = 96 \pm 2\%$.

the light at the grating outcoupler. This polarization configuration also helps in suppressing the residual laser scatter that does not couple to the waveguide at the input grating. By varying the position of the quarter-wave plate, we can vary the laser background in the setup. At each position of the waveplate, we measure the $g^{(2)}(0)$ and V_{raw}. Supplementary Fig. 6 shows the measured raw HOM visibility (plotted as $1 - V$) plotted against the measured $g^{(2)}(0)$. We fit the data to a first-order polynomial following Supplementary Eq. 3. The y-intercept is the intrinsic HOM visibility of the source. Using this approach, we estimate $V = 0.96 \pm 0.02$.

SUPPLEMENTARY NOTE 7. LONG-TERM OPERATIONAL STABILITY OF THE PLUG-AND-PLAY SOURCE

The long-term operational stability of the single-photon source is monitored by continuously measuring the generated photon rate over 110 hours. The detected count rate (normalized to the mean) over a 10 hour period is shown in Supplementary Fig. 7(a), which highlights that the source exhibits $< 2\%$ fluctuations in intensity (green shaded area). The residual slow variations in the count rates are primarily due to the long timescale thermal drifts in the single mode optical fibre that rotates the polarization of the single photons detected at the SNSPD. The SNSPD can exhibit up to 12% variation in the detection efficiency if the polarization of the single photons is changed from horizontal to vertical. Hence, we attribute the long term drifts in the count rates to the polarization drift, rather than the stability in fibre outcoupling from the device. Supplementary Figure 7(b) shows the intensity fluctuations, quantified as the ratio of standard deviation to the mean, of each 10 hour

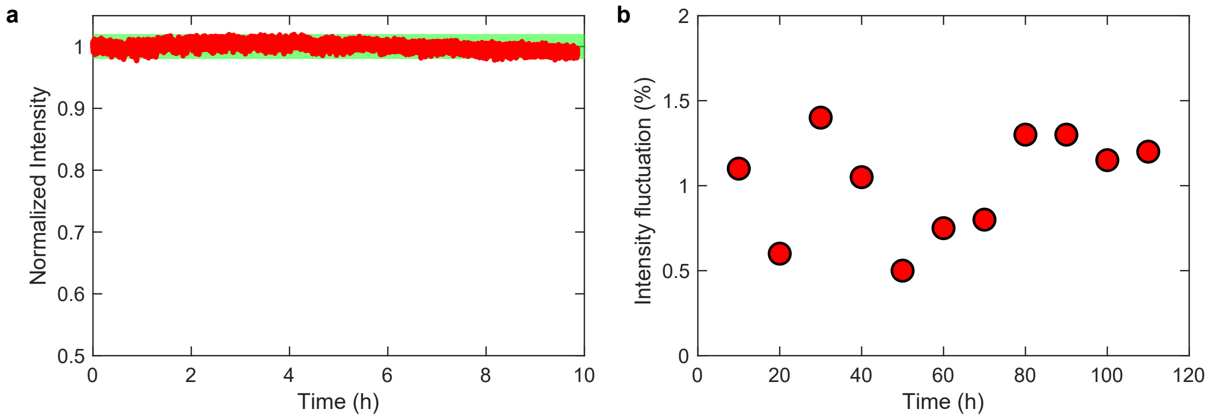

Supplementary Figure 7. (a) Detected single photon rate from the device over 10 hours of unmanned operation. The detected photon rate fluctuations are $< 2\%$ (green shaded area), most of which are due to the slow polarization variations in the single mode fibre relay to the superconducting nanowire single-photon detector (SNSPD). (b) Intensity fluctuations measured in eleven consecutive 10 hour acquisitions highlights stable long-term operation of the source with on-average $\approx 1\%$ intensity fluctuations.

acquisition over the 110 hour measurement period. On average, the intensity fluctuations are $\approx 1\%$ highlighting the stable long-term operation of the single-photon source.

SUPPLEMENTARY NOTE 7. THEORETICAL MODEL FOR RESONANCE FLUORESCENCE

We model the QD as a two-level system with ground state $|g\rangle$ and an excited state $|e\rangle$, with the frequency difference $\omega_{\rm qd}$. Defining the atomic raising and lowering operators $\hat{\sigma}_+ = |e\rangle\langle g|$ and $\hat{\sigma}_- = |g\rangle\langle e|$, respectively, we can write the non-interacting two-level system Hamiltonian as $\hat{H}_{\rm qd} = \hbar\omega_{\rm qd}\hat{\sigma}_+\hat{\sigma}_-$. We follow the derivation in [14] to calculate the resonance fluoresence signal from the QD. By driving the QD using a monochromatic field $\mathcal{E} = \mathcal{E}_0 e^{-i\omega_{\rm p}t}$, where $\omega_{\rm p}$ is the laser frequency that may be detuned from the QD by $\Delta = \omega_{\rm p} - \omega_{\rm qd}$, we can write the equation of motion for the resonantly excited QD with a radiative decay rate γ and dephasing rate $\gamma_{\rm d}$ as

$$\dot{\rho}(t) = \mathbf{M}\cdot\rho(t), \tag{4}$$

where

$$\mathbf{M} = \begin{pmatrix} 0 & i\Omega/2 & -i\Omega/2 & \gamma \\ i\Omega/2 & -\frac{\gamma+2\gamma_{\rm d}}{2} + i\Delta & 0 & -i\Omega/2 \\ -i\Omega/2 & 0 & -\frac{\gamma+2\gamma_{\rm d}}{2} - i\Delta & i\Omega/2 \\ 0 & -i\Omega/2 & i\Omega/2 & -\gamma \end{pmatrix} \tag{5}$$

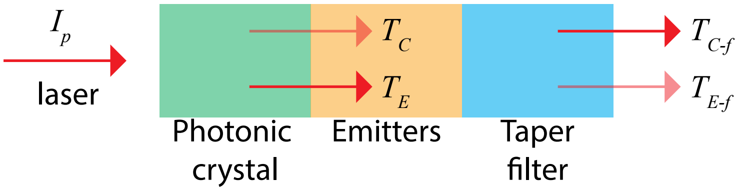

Supplementary Figure 8. Operational schematic of the nanophotonic structure highlighting the three essential sections: the photonic crystal, emitter, and the taper filter. The propagation of the excitation laser across the various sections in the two waveguide modes E and C are highlighted. The opacity of the arrows indicates the transmission of the modes; brighter arrow for a higher transmittance value.

and

$$\rho(t) = \begin{pmatrix} \rho_{\mathrm{gg}}(t) \\ \rho_{\mathrm{ge}}(t) \\ \rho_{\mathrm{eg}}(t) \\ \rho_{\mathrm{ee}}(t) \end{pmatrix}. \tag{6}$$

Here, Ω is the Rabi frequency, $\rho_{\mathrm{gg}}(t)$ and $\rho_{\mathrm{ee}}(t)$ are the ground and the excited state populations, respectively, and $\rho_{\mathrm{ge}}(t)$ and $\rho_{\mathrm{eg}}(t)$ denotes the coherence between the states.

Under pulsed resonant excitation of a QD with a Gaussian pulse, the Rabi frequency can be represented as

$$\Omega(t) = \frac{\Theta}{\sqrt{\pi}\sigma} e^{-(t-t_0)^2/\sigma^2}, \tag{7}$$

where, Θ is the pulse area related to the excitation intensity as $\Theta \propto \sqrt{I_{\mathrm{p}}}$ and σ is the half-width of the pulse. The time offset, t_0, is the center of the pulse.

We solve the system of differential equations (Supplementary Eq. 4) numerically for pulsed resonant excitation.

Derivation of the single photon impurity ξ

Supplementary Figure 8 illustrates the transport of the excitation laser with intensity I_{p} through the device. At the entrance of the photonic crystal section, the laser is equally coupled to the modes E (excitation) and C (collection) due to the Y-splitter design employed in the device shown in Fig. 1(b) of the main text. The photonic crystal filter transmits a fraction T_{E} of the mode E and T_{C} of the mode C. Typical values for T_{E} and T_{C} are shown in Supplementary Fig. 2. In the emitter section, the excitation laser propagates unperturbed up to the entrance of the taper filter section. The taper filter extinguishes the mode E while transmitting the mode C. The transmission of the modes E and C in the taper filter section are denoted $T_{\text{E-f}}$ and $T_{\text{C-f}}$, respectively.

The residual laser intensity I_{r} at the output is expressed as

$$I_{\mathrm{r}} = \frac{I_{\mathrm{p}}}{2}\left(T_{\mathrm{E}}T_{\text{E-f}} + T_{\mathrm{C}}T_{\text{C-f}}\right) \equiv I_{\mathrm{p}}T_{\mathrm{p}}. \tag{8}$$

The taper filters were numerically optimized to achieve $T_{\text{E-f}} \approx 10^{-6} - 10^{-7}$ and $T_{\text{C-f}} \approx 1$. Supplementary Fig. 2(b) shows that the photonic crystal suppresses the transmission of the exctation laser in the mode C with $T_{\mathrm{C}} \approx 10^{-5} - 10^{-6}$ at the operation wavelength of the device.

Under weak excitation of the QD, we can assume that the emitted single photon signal intensity I_{sp} is proportional to the excitation intensity I_{p}. We can express I_{sp} as

$$I_{\mathrm{sp}} = \frac{I_{\mathrm{p}}}{2}T_{\mathrm{E}}\beta_{\mathrm{E}}\left(\beta_{\mathrm{C}} + \beta_{\mathrm{E}}T_{\text{E-f}}\right) + \frac{I_{\mathrm{p}}}{2}T_{\mathrm{C}}\beta_{\mathrm{C}}\left(\beta_{\mathrm{C}} + \beta_{\mathrm{E}}T_{\text{E-f}}\right), \tag{9}$$

where we assumed $T_{\text{C-f}} = 1$. We can drop the last three terms as $T_{\text{E-f}}, T_{\mathrm{C}} \ll 1$ and express the collected single photon intensity as

$$I_{\mathrm{sp}} = \frac{I_{\mathrm{p}}}{2}T_{\mathrm{E}}\beta_{\mathrm{C}}\beta_{\mathrm{E}}, \tag{10}$$

which is the expression that we discuss in the main text. The impurity ξ can then be expressed by assuming $T_{\mathrm{E}} = 1$ as

$$\xi = \frac{I_{\mathrm{r}}}{I_{\mathrm{sp}}} \approx \frac{2T_{\mathrm{p}}}{\beta_{\mathrm{E}}\beta_{\mathrm{C}}} \tag{11}$$

Using the numerical solution of Supplementary Eq. 4 to extract the relation between the single photon emission and I_{p} in Supplementary Eq. 9, we can derive an exact expression for I_{sp} and ξ, which is shown in Fig. 3(b) of the main text.

SUPPLEMENTARY REFERENCES

[1] Löbl, M. C. *et al.* Excitons in InGaAs quantum dots without electron wetting layer states. *Commun. Phys.* **2**, 93 (2019).

[2] Midolo, L., Pregnolato, T., Kiršanskė, G. & Stobbe, S. Soft-mask fabrication of gallium arsenide nanomembranes for integrated quantum photonics. *Nanotechnology* **26**, 484002 (2015).

[3] Zhou, X. *et al.* High-efficiency shallow-etched grating on GaAs membranes for quantum photonic applications. *Appl. Phys. Lett.* **113**, 251103 (2018).

[4] Uppu, R. *et al.* Scalable integrated single-photon source. *arXiv:2003.08919* (2020).

[5] Pu, M., Liu, L., Ou, H., Yvind, K. & Hvam, J. M. Ultra-low-loss inverted taper coupler for silicon-on-insulator ridge waveguide. *Opt. Commun.* **283**, 3678–3682 (2010).

[6] Tiecke, T. *et al.* Efficient fiber-optical interface for nanophotonic devices. *Optica* **2**, 70–75 (2015).

[7] Uğurlu, A. D. *et al.* Suspended spot-size converters for scalable single-photon devices. *Adv. Quantum Technol.* **3**, 1900076 (2019).

[8] Tighineanu, P., Dreessen, C. L., Flindt, C., Lodahl, P. & Sørensen, A. S. Phonon decoherence of quantum dots in photonic structures: broadening of the zero-phonon line and the role of dimensionality. *Phys. Rev. Lett.* **120**, 257401 (2018).

[9] Iles-Smith, J., McCutcheon, D. P., Nazir, A. & Mørk, J. Phonon scattering inhibits simultaneous near-unity efficiency and indistinguishability in semiconductor single-photon sources. *Nat. Photon.* **11**, 521–526 (2017).

[10] Johansen, J., Julsgaard, B., Stobbe, S., Hvam, J. M. & Lodahl, P. Probing long-lived dark excitons in self-assembled quantum dots. *Phys. Rev. B* **81**, 081304 (2010).

[11] Davanço, M., Hellberg, C. S., Ates, S., Badolato, A. & Srinivasan, K. Multiple time scale blinking in InAs quantum dot single-photon sources. *Phys. Rev. B* **89**, 161303 (2014).

[12] Liu, F. *et al.* High purcell factor generation of indistinguishable on-chip single photons. *Nat. Nanotechnol.* **13**, 835–840 (2018).

[13] Kiršanskė, G. *et al.* Indistinguishable and efficient single photons from a quantum dot in a planar nanobeam waveguide. *Phys. Rev. B* **96**, 165306 (2017).

[14] Muller, A. *Resonance Fluorescence and Cavity Quantum Electrodynamics with Quantum Dots.* Ph.D. thesis, University of Texas at Austin (2007).
